# Generation of Potent Anti-Vascular Endothelial Growth Factor Neutralizing Antibodies from Mouse Phage Display Library for Cancer Therapy

**DOI:** 10.3390/ijms17020214

**Published:** 2016-02-05

**Authors:** Yan-Da Lai, Yen-Yu Wu, Yi-Jiue Tsai, Yi-San Tsai, Yu-Ying Lin, Szu-Liang Lai, Chao-Yang Huang, Ying-Yung Lok, Chih-Yung Hu, Jiann-Shiun Lai

**Affiliations:** Department of Protein Engineering, Development Center for Biotechnology, New Taipei City 22180, Taiwan; alienwhale@yahoo.com.tw (Y.-D.L.); liannyuyu@dcb.org.tw (Y.-Y.W.); yi_jiue@dcb.org.tw (Y.-J.T.); yisantsai@dcb.org.tw (Y.-S.T.); yuying1982@gmail.com (Y.-Y.L.); lucanus@dcb.org.tw (S.-L.L.); bchy@dcb.org.tw (C.-Y.H.); ziltoucan@dcb.org.tw (Y.-Y.L.); cyhu@dcb.org.tw (C.-Y.H.)

**Keywords:** phage display, single chain Fv antibody fragment (scFv), Fab antibody fragment, angiogenesis, vascular endothelial growth factor (VEGF)

## Abstract

Vascular endothelial growth factor (VEGF) is an important stimulator for angiogenesis in solid tumors. Blocking VEGF activity is an effective therapeutic strategy to inhibit tumor growth and metastasis. Avastin, a humanized monoclonal antibody recognizes VEGF, has been approved by the US Food and Drug Administration. To generate potential VEGF-recognizing antibodies with better tumor regression ability than that of Avastin, we have designed a systematic antibody selection plan. From mice immunized with recombinant human VEGF, we generated three phage display libraries, scFv-M13KO7, Fab-M13KO7, and scFv-Hyperphage, in single-chain Fv (scFv) or Fab format, displayed using either M13KO7 helper phage or Hyperphage. Solid-phase and solution-phase selection strategies were then applied to each library, generating six panning combinations. A total of sixty-four antibodies recognizing VEGF were obtained. Based on the results of epitope mapping, binding affinity, and biological functions in tumor inhibition, eight antibodies were chosen to examine their abilities in tumor regression in a mouse xenograft model using human COLO 205 cancer cells. Three of them showed improvement in the inhibition of tumor growth (328%–347% tumor growth ratio (% of Day 0 tumor volume) on Day 21 *vs.* 435% with Avastin). This finding suggests a potential use of these three antibodies for VEGF-targeted therapy.

## 1. Introduction

Monoclonal antibody drugs have been shown to be one of the powerful disease treatments, including for cancers. One of the important strategies is applying monoclonal antibodies to block tumor survival signals, including cell proliferation and angiogenesis. Angiogenesis is a fundamental physiological process of new capillaries sprouting and remodeling from pre-existing blood vessels. The angiogenic process is complex and involves a delicate balance between many pro-angiogenic and anti-angiogenic factors, as well as different cell types [1,2,3,4]. It has been well established that angiogenesis plays a role in pathological conditions, especially on tumor proliferation and metastasis. The efficacy of chemotherapy is also decreased in cancer patients with angiogenesis [5,6]. Tumor angiogenesis is characterized by abnormal vessel formation and high level of vascular endothelial growth factor (VEGF) in tumor microenvironments.

VEGF, a 45-kDa homodimeric glycoprotein specific for vascular endothelial cells, is critical for vasculogenesis, lymphangiogenesis and angiogenesis. In human, the VEGF family includes VEGF-A, VEGF-B, VEGF-C, VEGF-D, and placenta growth factor. All VEGF members-mediated cellular responses are through binding to VEGF receptors (known as VEGFR1, VEGFR2 and VEGFR3) on the cell surface. After ligand binding, the VEGFRs are activated by trans-phosphorylation. VEGF-A binds to VEGFR1 and VEGFR2; VEGFR2 is believed to play the primary role in mediating most known cellular responses to activate endothelial cells. Several VEGF-A variants with different biological properties can be generated by alternative splicing as follows: VEGF-A_121_, VEGF-A_145_, VEGF-A_165_, VEGF-A_183_, VEGF-A_189_, and VEGF-A_206_. In human, VEGF-A_165_ is the predominant variant and commonly referred to as VEGF.

Previous studies have shown that the VEGF pathway is involved in tumor angiogenesis. Therefore, inhibiting VEGF signaling to block the growth of tumor cells, including neutralizing antibodies against VEGF or VEGFR and tyrosine kinase inhibitors against VEGFR, has been developed as therapeutics [7,8,9,10]. The humanized anti-VEGF monoclonal antibody bevacizumab (Avastin^®^) is the first anti-angiogenic agent approved by the US Food and Drug Administration as a treatment for different types of tumor in combination with chemotherapy [11,12,13]. The clinical success of Avastin greatly encourages the development of targeted therapy against the VEGF pathway.

To isolate VEGF-neutralizing antibodies recognizing epitopes different from the unveiled Avastin binding epitopes on VEGF [14], we applied phage display system to generate comprehensive anti-VEGF antibody repertoires. This technology offers a way to overcome the limitations of hybridoma technology [15,16,17] and increases the posibility of isolating high affinity antibody candidates after systematic library screening procedure. In this work, we constructed single-chain Fv (scFv) and Fab phage libraries from mice immunized with human VEGF, and displayed them with M13KO7 helper phage (scFv and Fab library) or Hyperphage (scFv library only) to generate three library-display combinations. Two selection methods were then applied individually on these libraries to generate six panning combinations. Sixty-four antibodies were isolated, and eight of them were fully characterized via epitope mapping, *in vitro* analyses of the inhibition ability in tumor cell proliferation and migration, and the phosphorylation of VEGFR2. The *in vivo* antitumor efficacy was also evaluated in the growth of COLO 205 human colon cancer cell tumor xenografts in an athymic nude mouse model system. A detailed comparison between selected anti-VEGF clones and Avastin was investigated.

## 2. Results

### 2.1. Selection of Anti-VEGF Antibody Fragments from Phage Display Libraries

To generate phage library with enriched antibodies against VEGF, mRNA was isolated from the spleen cells of BALB/c mice immunized with recombinant human VEGF and used to construct scFv and Fab phage display libraries, which were then displayed on M13KO7 helper phage (scFv and Fab libraries) or Hyperphage (scFv library) producing three library-display combinations. The scFv library has 3.12 × 10^9^ individual members and the Fab library contains 1.02 × 10^9^ individual members. ScFv and Fab antibodies recognizing human VEGF in the above-mentioned three library-display combinations were then selected individually using solid-phase (immunoplate) or solution-phase (Dynabeads) panning methods resulting in six combinations of panning methods, including Fab-KO7-immunoplate, scFv-KO7-immunoplate, scFv-Hyperphage-immunoplate, Fab-KO7-beads, scFv-KO7-beads, and scFv-Hyperphage-beads. After three to six rounds of selection, 800–1600 random colonies per panning strategy were picked, and the corresponding monoclonal phages were prepared to examine the binding abilities of VEGF by phage enzyme-linked immunosorbent assay (ELISA). Their heavy chain variable region (VH) and heavy chain variable region (VL) nucleotide sequences were also determined. As shown in Table 1, seven to fourteen clones per panning method combination were chosen and a total of sixty-four unique scFv or Fab phage binders recognizing VEGF were obtained.

**Table 1:** Binding affinity, epitope and anti-proliferation activity of isolated clones.

**Fab-KO7-Immunoplate**					**Fab-KO7-Beads**				
**Clone**	**Epitope**	**IgG ELISA *K*_D_ (nmol/L)**	**Fab ELISA *K*_D_ (nmol/L)**	**EC_50_ ng/mL**	**Clone**	**Epitope**	**IgG ELISA *K*_D_ (nmol/L)**	**Fab ELISA *K*_D_ (nmol/L)**	**EC_50_ ng/mL**
2F8	D	3.34	502.0	-	BA2	BD	243.40	>10,000	N.A.
2H11	D	0.64	784.0	-	B-C4	D	0.13	604.0	-
2D8	D	0.84	2500.0	-	BC9	D	0.09	97.1	23,325
1A3	BD	2.28	1911.0	-	BD2	D	0.16	49.8	207
1D1	D	22.60	898.0	N.A.	BE4	D	0.08	146.0	3242
2G10	D	1.40	>10,000	N.A.	BC11	BCD	5.67	58.5	9677
4-4B4	N.A.	-	-	N.A.	B-F10	D	26.60	-	4945
4-4C6	N.A.	-	-	N.A.					
4H6	A	0.04	373.0	-					
3E9	A	3.42	3000.0	-					
1E7	D	0.40	1650.0	-					
1A5	D	0.30	732.0	6744					
**ScFv-KO7-Immunoplate**					**ScFv-KO7-Beads**				
**Clone**	**Epitope**	**IgG ELISA *K*_D_ (nmol/L)**	**ScFv ELISA *K*_D_ (nmol/L)**	**EC_50_ ng/mL**	**Clone**	**Epitope**	**IgG ELISA *K*_D_ (nmol/L)**	**ScFv ELISA *K*_D_ (nmol/L)**	**EC_50_ ng/mL**
K2A3	BD	0.16	23.1	5438	BK3C1	ABD	0.11	N.A.	-
K3-2D4	ACD	0.15	168.0	-	BK3A10	ABCD	0.56	N.A.	-
K3H5	Δ	23.92	114.0	N.A.	BK3A3	D	0.06	N.A.	9.9
K4H8	AB	5.45	31.2	6188	BK3C2	Δ	1355.00	N.A.	N.A.
K1D9	ABD	1.72	29.0	-	BK3B9	D	0.12	N.A.	N.A.
K3-2H2	BD	0.16	1927.0	N.A.	BK3A5	BCD	0.18	N.A.	-
K3-2H8	D	0.09	20.7	2802	BK3D6	D	9.47	N.A.	N.A.
K1D1	AD	2.94	1640.0	7712	BK3D7	ABCD	7.20	N.A.	N.A.
K3-2F3	Δ	28.30	N.A.	N.A.	BK3C12	BCD	1.80	N.A.	175.0
K3H6	C	0.05	16.7	-	BK3A9	BD	3.40	N.A.	-
K3H2	BD	0.12	49.4	3472	BK3B1	ABCD	0.12	N.A.	97.1
K3-1B1	Δ	0.13	12.4	241					
K3-3B1	A	190.00	641.0	N.A.					
K1D10	Δ	950.00	64.0	N.A.					
**ScFv-Hyperphage-Immunoplate**					**ScFv-Hyperphage-Beads**				
**Clone**	**Epitope**	**IgG ELISA *K*_D_ (nmol/L)**	**ScFv ELISA *K*_D_ (nmol/L)**	**EC_50_ ng/mL**	**Clone**	**Epitope**	**IgG ELISA *K*_D_ (nmol/L)**	**ScFv ELISA *K*_D_ (nmol/L)**	**EC_50_ ng/mL**
H3-2G1	A	16.63	3230.0	N.A.	BH3F6	Δ	2.16	N.A.	-
H3-2A8	ABD	2.74	3000.0	-	BH3B4	Δ	0.47	N.A.	101.0
H3-3C7	N.A.	75.30	1290.0	N.A.	BH3A12	BCD	2.52	N.A.	22.6
H3-3C12	Δ	4.12	47.1	-	BH3G12	D	0.06	N.A.	27.9
H3-3H7	D	431.00	11,900.0	N.A.	BH3B5	D	0.13	N.A.	-
H3-3H6	A	100.00	117.0	N.A.	BH3D4	BD	0.07	N.A.	31.0
H3-1A3	A	1.80	174.0	N.A.	BH3F5	D	0.05	N.A.	42.0
H3-1B1	Δ	0.14	641.0	9.6 *	BH3F11	ABD	0.23	N.A.	-
H3-2A9	Δ	0.14	27.8	5361.0	BH3F7	AB	0.04	N.A.	-
H3-3E1	Δ	0.07	32.8	-	BH3C3	Δ	0.18	N.A.	4.8

Abbreviations: EC_50_, the half maximal effective concentration; N.A., not available; -, no inhibitory effect in VEGF-induced HUVEC proliferation assay; *, atypical proliferation curve; Δ, the epitope is not located in cluster A, B, C or D. The A, B, C, and D are the binding epitopes toward VEGF as described in results.

### 2.2. Binding Affinity of Anti-VEGF Antibodies

To evaluate the binding affinities of scFv or Fab fragment antibodies, DNA fragments containing the variable regions of the forty-five scFv-phage binders were subcloned into pET-27b(+) expression vector for the production of scFv antibodies. Nineteen Fab clones expressed in the phagemid pCOMB3X needed no extra subcloning processes for the expression of the soluble Fab antibodies. The average binding affinity of the twenty-four first-subcloned scFvs and seventeen Fab fragment antibodies were unexpectedly low (ranging from 12.4–11,900 nM, Table 1). Fab clones 4-4B4 and 4-4C6 showed low expression level (data not shown). The scFv and Fab antibodies of Avastin also showed unexpectedly dramatic decrease in their binding affinities against VEGF (data not shown). To avoid the possibility of the subtle protein folding differences between full-length antibody and scFv/Fab that might affect VEGF binding, all of the clones were converted into full-length chimeric antibody format to confirm their binding affinities. Interestingly, the binding *K*_D_ measured by ELISA showed that the binding affinities of most full-length antibodies were stronger than their corresponding scFv and Fab fragments except scFv clone K1D10 (columns 3 and 4 in Table 1). For example, 0.04 *vs.* 373 nM in clone 4H6 selected by Fab-KO7-immunoplate panning method or 0.13 *vs.* 12.4 nM in clone K3-1B1 selected by scFv-KO7-immunoplate method. Therefore, the remaining twenty-one scFv clones were directly converted to full-length antibody format to determinate their binding affinity against VEGF. Forty-one out of sixty-four full-length antibodies were used to investigate the antibody effects on VEGF-induced receptor phosphorylation, cell proliferation and migration.

### 2.3. Epitope Mapping of Anti-VEGF Antibodies

**Figure 1:**
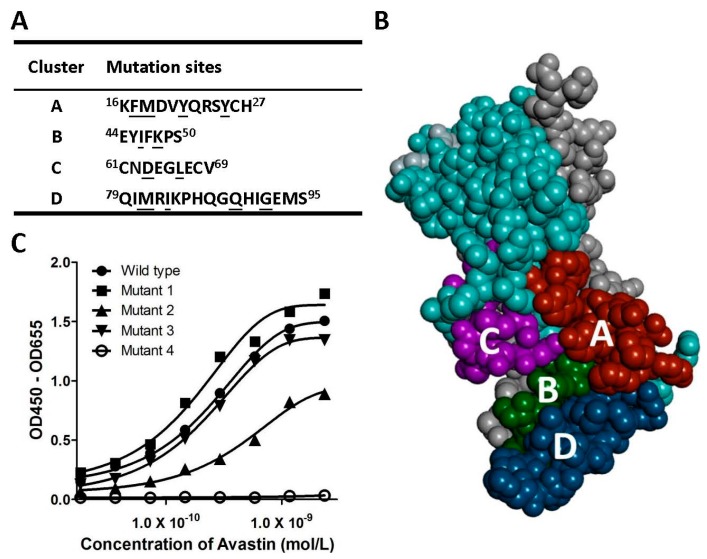

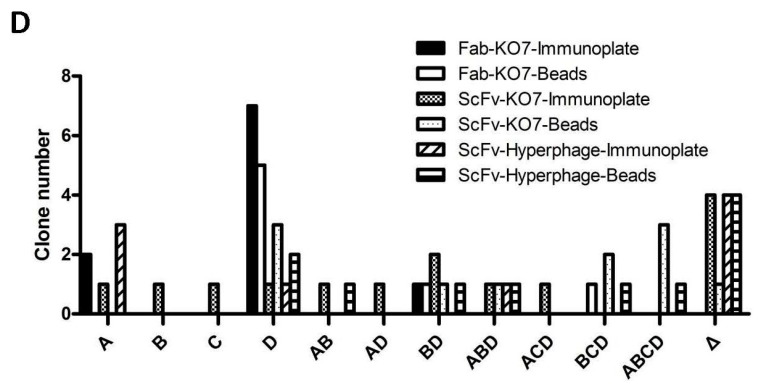
Epitope mapping of isolated antibodies. (**A**) The amino acid sequences of the four epitope clusters on VEGF and the selected residues for alanine-scanning. (**A**–**D**) Epitope clusters chosen for generating VEGF mutants. The key contact residues (underlined) of VEGF were replaced by alanine. The mutant and the wild type VEGFs were used to map the epitopes; (**B**) The three-dimensional structure of VEGF dimer and the interaction surface with VEGFR2. The VEGF monomers are shown in cyan and gray, respectively. Four clusters (**A**–**D**) of the receptor binding surface are colored in brown, green, purple, and blue, respectively. Clusters **A** and **C** are located on one monomer (cyan) and the clusters **B** and **C** are located on the other one (gray); (**C**) The Avastin binding to four VEGF mutants. Various concentrations of Avastin bound to mutant 1, mutant 2, mutant 3, and mutant 4, and the kinetics against these VEGF variants were determined by ELISA. OD, optical density; (**D**) The analysis of the number of isolated clones by different selection strategies and their VEGF epitope clusters.

In order to elucidate the epitopes of VEGF recognized by selected antibodies, alanine-scanning approach was used to identify the critical residues on the surface of VEGF. The key residues of VEGF that interact with VEGF receptors include F17, I46, E64, Q79, and I83 for VEGFR2 binding and F17, M18, Y21, Y25, K48, D63, L66, M81, and I83 for VEGFR1 binding [18,19]. Another study has also shown the importance of M81, Q89, and G92 in the Avastin–VEGF interaction [14]. Therefore, we grouped these residues into four clusters (clusters A–D, sequences are underlined in Figure 1A) and mutated them into alanines for epitope mapping. The four mutants of VEGF covering cluster A, B, C or D were referred to as mutant 1 (F17A, M18A, Y21A, and Y25A), mutant 2 (I46A and K48A), mutant 3 (D63A and L66A), and mutant 4 (M81A, I83A, Q89A, and G92A). Figure 1B shows the relative location of the four chosen clusters on the three-dimensional structure of antiparallel VEGF homodimer (see figure legend for detail).

The binding affinities of the anti-VEGF antibodies against VEGF mutants were measured by ELISA. Figure 1C shows the result of epitope mapping using Avastin as an example. The binding activity between Avastin and mutant 4 was completely abolished; however, the binding of Avastin to mutant 1, mutant 2, and mutant 3 remained insignificantly changed (Figure 1C). This result indicated that Avastin binding site is located in the D region, which is consistent with a previous report [14]. We also analyzed the VEGF epitopes of the sixty-two antibodies isolated from the six panning method combinations. Figure 1D shows the summarized result of epitope mapping; epitope clusters are grouped as one singe cluster (A, B, C, or D), combinations of two clusters (AB, AD or BD), three clusters (ABD, ACD, or BCD), or all four clusters (ABCD). A total of twenty-seven, nine, nine, and four scFv or Fab clones recognized across one, two, three, and four epitope clusters, respectively, were observed. The isolated clone numbers of each group were similar (one to six clones per group), however, the numbers of antibodies recognizing cluster D were more than all the other epitope clusters (nineteen clones). Interestingly, the epitopes of the thirteen clones were not located in any of the four clusters mentioned above. The epitope of each selected clone is listed in Table 1.

### 2.4. Anti-VEGF Antibodies Inhibit Human Umbilical Vein Endothelial Cell (HUVEC) Proliferation

**Figure 2:**
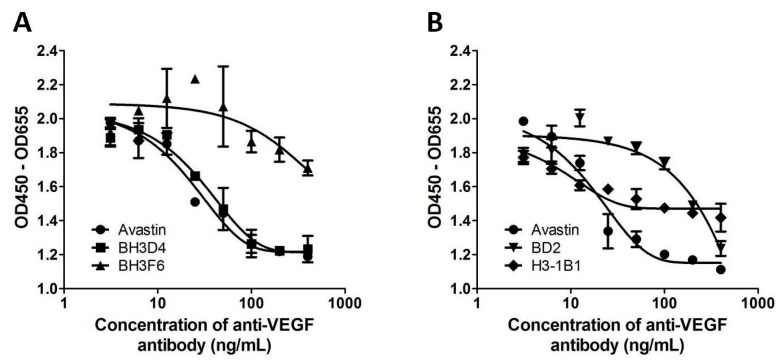
Proliferation assay: Inhibition of VEGF-induced cell proliferation. Avastin and test antibodies were serial diluted and added to the 96-well tissue culture plates. The antibody was mixed with VEGF and incubated at room temperature for 30 min. HUVEC cells were harvested and added to each well. After incubation, WST-1 was added to determine the absorbance at 450 and 655 nm. (**A**) An example of anti-proliferation effects (BH3D4); (**B**) An example of an atypical curve (H3-1B1).

To further confirm the biological activities of these isolated antibodies, forty-one out of sixty-two antibodies were selected for proliferation assay based on the results of epitope analysis and ELISA binding assay. In Figure 2A, the EC_50_ of Avastin and BH3D4 is 20 and 31 ng/mL, respectively, and BH3F6 shows no inhibitory activity. In Figure 2B, H3-1B1 shows an atypical proliferation curve but its calculated EC_50_ was 9.6. The EC_50_ result of each clone is summarized in Table 1. A total of seven antibodies selected from the scFv library showed comparable activity to Avastin in VEGF-induced proliferation. These seven antibodies and BD2, the only one antibody acting more effectively from the Fab library, were subjected to further analyses.

### 2.5. Anti-VEGF Antibodies Suppress VEGF-Induced VEGFR2 Phosphorylation and HUVEC Migration

**Figure 3:**
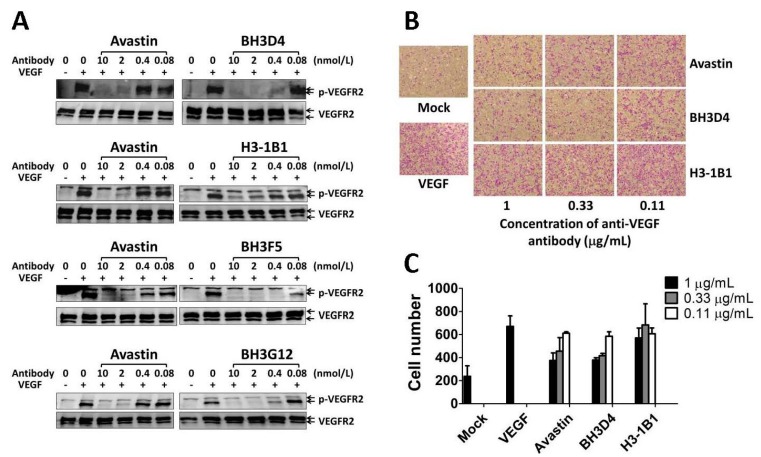
Inhibition of VEGF-induced cell migration. (**A**) Inhibition of VEGF-induced VEGFR2 phosphorylation. HUVECs were starved and treated with VEGF and test antibodies. Cell lysates were electrophoresed and analyzed by Western blot with anti-VEGFR2 and anti-phospho-VEGFR2 (Tyr1175) antibody; (**B**) The migration activity of HUVECs was evaluated using a Transwell assay system. Cells were added onto the top chamber, whereas migration stimuli VEGF mixed with various concentrations of the test antibodies were added to the lower chamber. After 22 h of incubation, the cells on the lower face of the membrane were stained and counted under microscope at 100× magnification; (**C**) The cell counts were obtained from three random fields and presented as mean ± SD.

VEGF binding to its receptor induces receptor dimerization and autophosphorylation of tyrosine residues in the intracellular kinase domain. Binding of PLC-γ to the phosphorylated Y1175 (pY1175) of VEGFR2 results in cell proliferation, whereas binding of Shb to pY1175 regulates the activation of cell migration [20]. To assess the capability of antibody to inhibit the VEGF-induced VEGFR2 phosphorylation, a serial dilution of these eight neutralizing antibodies was used to test in HUVECs at the concentrations of 0.08, 0.4, 2, 10, and 50 nM. As shown in Figure 3A, BH3D4, BH3F5 and BH3G12 significantly inhibited the phosphorylation of VEGFR2 at the concentration of 0.4 nM, which was five-fold more effective than Avastin (2 nM). Additionally, H3-1B1 had only slight inhibition effect at 10 nM. The summarized result of these eight antibodies is tabulated in Table 2. The inhibitory effect of antibodies BH3D4, BD2, BH3F5, BK3A3, BH3C3, and BH3G12 was at least five-fold more effective than Avastin, but BH3C3 was not dose-dependent (data not shown). Antibodies BH3A12 and H3-1B1 showed less inhibition activity.

To investigate the inhibition effect of the eight anti-VEGF antibodies on the migration of cells under the VEGF treatment, HUVECs were allowed to migrate through the membrane of the Transwell assay system. Various concentrations of antibody-VEGF mixture were used as migration stimuli. The number of cells traversing the chamber membrane was counted and analyzed (Figure 3B). BH3C3 had a slight effect, whereas BH3A12 and H3-1B1 showed no inhibitory activity on HUVEC migration. The abilities of BH3D4, BD2, BH3F5, BK3A3 and BH3G12 to inhibit cell migration were similar to Avastin (Figure 3C and Table 2).

**Table 2:** Summary of top eight positive clones with VEGF-neutralizing activity in proliferation, phosphorylation and migration assays.

Clone	Epitope	ELISA	BIAcore			Anti-Poliferation EC_50_ (ng/mL)	Anti-Phosphorylation ^a^	Anti-Migration ^b^	Tumor Growth Ratio (%) on Day 21
		*K*_D_ (nmol/L)	*K*_on_ (×10^6^ M^−1^·S^−1^)	*K*_off_ (×10^−3^ S^−1^)	*K*_D_ (nmol/L)				
BD2	D	0.16	4.64	0.98	0.21	207.0	+++	++	540 ± 339
BH3D4	BD	0.07	5.98	1.77	0.30	31.0	+++	++	328 ± 61
BH3F5	D	0.77	11.90	2.79	0.23	42.0	+++	++	462 ± 137
BK3A3	D	0.06	4.32	1.45	0.34	9.9	+++	++	340 ± 161
BH3G12	D	0.06	5.37	1.41	0.26	27.9	++	++	347 ± 108
BH3A12	BCD	2.52	11.90	16.30	1.31	22.6	+	-	533 ± 189
H3-1B1	Δ	0.14	N.A.	N.A.	N.A.	9.6 *	+	-	504 ± 60
BH3C3	Δ	0.18	5.36	1.45	0.27	4.8	+++ *	+	513 ± 271
Avastin	D	0.05–0.2	18.00	3.94	0.22	20.0	++	++	435 ± 133

N.A., not available; *, atypical effect curve; Δ, the epitope is not located in cluster A, B, C or D. The A, B, C, and D are the binding epitopes toward VEGF as described in results; ^a^ Anti-phosphorylation activity: +++, effect dose level of 0.4–0.08 nmol/L; ++, 2.0–0.40 nmol/L; +, 10.0–2.00 nmol/L; ^b^ Anti-migration activity: ++, effect of Avastin or comparable to Avastin; +, effect lower than Avastin; -, no inhibitory effect; Tumor Growth Ratio (%), % of Day 0 tumor volume, Growth (%) ± SD, *n* = 5.

### 2.6. Affinity Measurement and Kinetic Analysis Using BIAcore

The Surface Plasmon Resonance (SPR) based assay with Biacore T100 was applied to measure the affinities and binding kinetics of VEGF-neutralizing antibodies. The binding kinetics was measured and analyzed by multi-cycle kinetics methods. The VEGF binding affinities of BH3C3, BH3G12, BK3A3, BH3F5, BH3D4, BH3A12 and BD2 ranged from 0.21 to 1.31 nM, which were similar to that of Avastin (0.22 nM) (Table 2). The binding kinetics of H3-1B1 was not determined but was verified on its stable pool-expressed antibody. The affinity of stable pool-expressed H3-1B1 was ten-fold less than the other seven stable pool-expressed VEGF-recognizing antibodies (data not shown).

### 2.7. In Vivo Tumor Growth Inhibition Assay in Colorectal Tumor Xenograft Model

**Figure 4:**
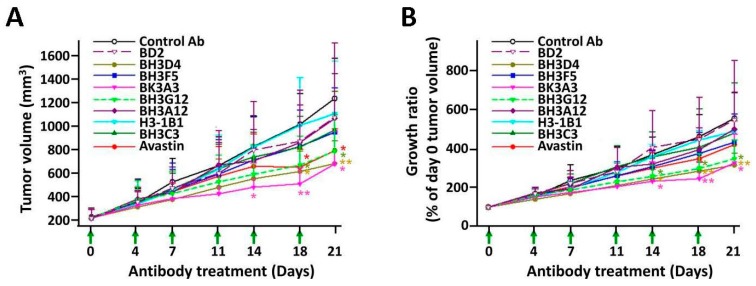
Tumor growth inhibition experiment in xenograft model. (**A**) Antitumor activity on human COLO 205 cancer cell xenografts of Avastin and test antibodies were measured by Day 21 tumor volume. Anti-VEGF antibodies were administered intraperitoneally (i.p.) twice per week for three consecutive weeks. Data points indicate mean ± SD of tumor volume (*n* = 6, except *n* = 5 in Avastin group). The statistical analysis was performed by Student *t*-test. The single star represents *p* < 0.05, whereas the double star represents *p* < 0.01; (**B**) The trend of tumor growth ratio. Anti-VEGF antibodies at the concentration of 0.1 mg/kg were administered i.p. twice per week for three weeks. Data points indicate mean ± SD of tumor growth ratio (*n* = 6, except *n* = 5 in Avastin group). The statistical analysis was performed by Student *t*-test. The single star represents *p* < 0.05, whereas the double star represents *p* < 0.01.

VEGF is essential for tumor growth and metastasis. The *in vitro* assays demonstrated that our anti-VEGF antibodies are sufficient in inhibiting various biological function of VEGF. Therefore, we speculated that they should also be potent in suppressing tumor growth and metastasis *in vivo*. Mouse xenograft study using human COLO 205 cancer cell was performed to determine the efficacy of anti-VEGF antibodies in five-week-old BALB/cAnN.Cg-Foxnlnu/CrlNarl nude mice. When tumors reached an average volume greater than 150 mm^3^, the mice were randomly grouped and treated with Avastin or test antibodies at the dosage of 0.1 mg/kg twice a week for three weeks. Figure 4A shows the result of tumor volume over the test period (21 days) for various treatment groups. The tumor growth ratio of mice treated with test antibodies is shown in Figure 4B. Noticeably, BH3D4, BK3A3, and BH3G12 showed significantly stronger inhibition effect than Avastin (Day 21 tumor growth ratio, expressed as % of Day 0 tumor volume, of 328%, 340%, and 347% *vs.* 435%, respectively). The experiments were repeated three times and showed similar results (data not shown). The angiogenesis intensity was measured by microvessel density examination with CD34 (the marker of vessel endothelial cells) immunochemistry staining of sections from fixed-tumor tissues. The CD34-positive area ratios of BH3A12, BH3D4, BK3A3, and BH3G12 were significantly greater reduced than Avastin after drug treatment (51%, 58%, 74%, 50% *vs.* 32% reduction, respectively, unpublished results).

## 3. Discussion

Many studies have shown the importance of VEGF and its receptors in tumor angiogenesis. The inhibition of VEGF-mediated angiogenesis and the formation of new blood vessels by specific monoclonal antibody or other inhibitors shows more advantageous in the cancer therapies [21]. Phage display has been shown as one of the most widely applied technologies capable of generating new therapeutic antibody drugs over the past two decades [22,23,24,25]. In addition, it is highly efficient for the generation of antibody from phage display library made by immunized mouse [26]. In this study, we obtained sixty-four antibodies against VEGF from the immunized mouse scFv and Fab phage display libraries. Seven antibodies have shown comparable cell proliferation inhibition ability to that of Avastin. Furthermore, *in vivo* anti-tumor activities of these seven antibodies were also determined in mouse xenograft model and three of them showed better tumor inhibition than Avastin.

To isolate a great number of hits against VEGF, M13KO7 helper phage was used for scFv and Fab phages preparation, and Hyperphage was used for scFv library preparation. These three library-display combinations were then individually selected by immunoplate and Dynabeads methods. Interestingly, only one antibody, H3-1B1, was isolated by using the immunoplate method and the other seven antibodies were isolated by using the Dynabeads method. In addition, two of the eight antibodies were selected from the scFv or Fab library displayed on M13KO7 helper phage (BK3A3 and BD2) and the other six were selected by using Hyperphage system (Table 1). Taken together, we found that five out of eight antibodies were obtained via solution-phase panning combined with scFv-Hyperphage display method.

Both solid-phase panning through immunoplate or solution-phase panning through Dynabeads were reported in previous studies [27,28]. In immunoplate-based panning, the epitope may be partially denatured after immobilization which may lead to the difficulty in the selection of antibodies which recognize the native antigen [29]. In contrast, in Dynabeads method the biotin-labeled native antigen in solution is free to expose to the phage library [30,31]. Consistent with our experiences, Dynabeads method is more advantageous for selecting effective antibodies than immunoplate method, although Dynabeads method is less convenient to operate.

Because hyperphage lacks helper phage-encoded gene 3, the phagemid-encoded g3p fusion antibody is the only source of g3p in virion assembly. The antigen-binding activity is enhanced by increasing the number of antibodies displayed per phage particle. Thus, the Hyperphage system can perform with small amount of antigen and increase panning efficiency [32]. Dynabeads–Hyperphage system is more suitable for selection of our target protein in light of the results of this study.

Comparing to the full-length antibody, scFv and Fab are smaller and easier to quickly engineer and produce recombinant antibody fragment for further experiment [29]. Thus, these two formats are commonly chosen for antibody production and binding affinity analysis. Surprisingly, we found the VEGF-binding affinities of our selected antibodies in scFv or Fab format were significantly lower than the full-length antibody format (Table 1), and this decrease in affinity may due to the monovalent binding [33].

An ideal anti-VEGF antibody in clinics is to interrupt the interaction between the binding surface of VEGF and VEGF receptors. In this study, the epitope recognized by BD2, BK3A3, BH3F5 and BH3G12 were similar to which targeted by Avastin and all of them were located in cluster D on VEGF. On the other hand, the epitopes binding by BH3D4, BH3A12, H3-1B1 and BH3C3 were different from Avastin’s binding region. The epitope for BH3D4 binding might be B and D clusters and the epitope for BH3A12 recognition might locate in three clusters B, C, and D. Interestingly, H3-1B1 and BH3C3 remain the binding affinity to all four VEGF mutants and it indicates that the epitopes for these two antibodies were not located in the clusters A, B, C, and D (Table 2).

In addition to epitope binding assay, the antibodies from our screening results were further used to test the ability of tumor growth inhibition. Based on the results of *in vitro* and *in vivo* tumor inhibition assays, BD2, BH3D4, BH3F5, BK3A3 and BH3G12 were effective in inhibiting tumor growth. Interestingly, these data were correlated to epitope mapping results. BD2, BH3F5, BK3A, and BH3G12 bound to the epitopes, which were also recognized by Avastin. Based on these observations, the different antibody clones, which recognize similar epitopes in the same antigen, may have comparable biological functions, such as tumor regression. Moreover, we also observed that the H3-1B1 and BH3C3 epitopes were not located in clusters A, B, C, and D and showed slightly effective on cell migration assay and *in vivo* mouse xenograft study. However, BH3A12 had strong binding affinity against VEGF and its epitope includes three clusters (B, C, and D), but it had only modest inhibitory effect (Table 2). This controversial result may be due to the ten-fold less binding affinity against VEGF comparing to the other seven anti-VEGF antibodies.

It is interesting that even though we used a large mouse library of more than 1 × 10^9^ individual members, all antibodies with better capability in neutralizing VEGF in this study had a very similar epitope region as Avastin. Mouse VEGF is very similar to human VEGF and only few amino acid difference between human and mouse are located in the VEGF active site. Most of the immunogenicity will be from those different amino acid sequence of human and mouse VEGF if hybridoma is applied for antibody generation. Avastin and our antibodies are generated by the same approach, which is why our antibodies recognized similar epitopes to Avastin. In addition, we also found that the cluster D was significantly more immunogenic than the other three clusters (Figure 1D). Accordingly, this cluster was easier to produce neutralizing antibodies. For example, a total of nineteen clones specifically recognized cluster D, whereas only one to six clones specifically recognized cluster A, B, or C.

VEGF is known for its angiogenic property. Many pharmaceuticals have been developed based on the theory that blocking angiogenesis can inhibit cancer cells. These pharmaceuticals include Avastin, 2C3 [34,35,36], IMC-1121 [34,37], VEGF-Trap [34] and small molecule inhibitors [38,39]. Some of them have emerged on the market and have been therapeutic options to improve the survival rate or the quality of life in patients. In this study, we have generated three VEGF-neutralizing antibodies (BH3D4, BK3A3, and BH3G12) and demonstrated their ability in the inhibition of VEGF-induced receptor phosphorylation, migration and proliferation on endothelial cells. Furthermore, all were comparable or even better than Avastin in tumor xenograft model. Therefore, these three antibodies have provided the potential for VEGF-targeted therapy.

Recently, X-ray crystallography studies have shown that rituximab and obinutuzumab (GA101) recognize a partially overlapping epitope on CD20, but differ fundamentally in their biological properties. Both of them have been approved for the treatment of chronic lymphocytic leukemia [40,41,42,43] implying the clinical potential of new candidates recognizing epitopes in proximal positions. In accordance with this observation, our anti-VEGF antibodies shared overlapping epitope with Avastin, but the result of mouse xenograft study showed they were different from Avastin. This suggests the possibility that our anti-VEGF antibodies and Avastin have distinct biological functions. These antibodies have the potential to be developed as therapeutic agents. Moreover, with the epitope characteristics of these VEGF neutralizing antibodies, the cluster D is an adequate epitope to generate the neutralizing antibodies from the immunized mouse.

## 4. Experimental Section

### 4.1. Immunization, RNA Extraction and cDNA Synthesis

Six-to-eight-week-old BALB/c mouse was injected four times with recombinant human VEGF (R&D, Minneapolis, MN, USA). The mouse received 15, 10, 12.5, and 10 μg of VEGF on Days 0, 14, 28 and 42, respectively. The immunogen was emulsified with Freund’s complete adjuvant (Day 0) (Sigma, St. Louis, MO, USA) or Freund’s incomplete adjuvant (Days 14, 28 and 42) (Sigma) subcutaneously. Blood samples were collected 10–14 days after each boost to monitor the immune response against VEGF using ELISA. On Day 56, the spleen was harvested and homogenized by TRIzol reagent (Life Technologies, Carlsbad, CA, USA) according to the manufacturer’s instructions. RNA from the spleen was reverse-transcribed with oligo d(T)18 primer (NEB, Ipswich, MA, USA).

### 4.2. Construction of scFv Phage Display Library

The scFv construct was assembled by two-step overlap extension polymerase chain reaction (PCR). Pairs of published oligo primers [44] were used to amplify VH, (G_4_S)_3_ linker and VL fragments from cDNA described above. A mixture VH, linker and VL fragments were mixed first for a 30-cycle PCR reaction without primer to join the fragments together by using Advantage 2 Polymerase Mix (Clontech, Mountain View, CA, USA). The reaction mixture was further subjected to amplification for 30 cycles using primer pairs carrying *Sfi*I and *Not*I restriction sites [44] for the directional cloning into the phagemid vector pCANTAB5E (Amersham Biosciences, Little Chalfont, Buckinghamshire, UK). Ligation mixture was electroporated into *Escherichia coli* (*E. coli*) TG1 (Lucigen, Middleton, WI, USA).

### 4.3. Construction of Fab Phage Display Library

Mouse heavy (Fd) and light chain cDNAs were PCR-amplified using specific primer pairs with *Xho*I/*Spe*I (Fd) or *Sac*I/*Xba*I (light chain) restriction sites [45]. First, the light chain insert (*Sac*I-*Xba*I) was cloned into pComb3X phage display vector. The resulting plasmid containing light chain gene repertoire was subjected to heavy chain cloning (*Xho*I-*Spe*I). The final construct containing both the light chain and heavy chain DNA repertoire was subsequently transformed into TG1.

### 4.4. Preparation of Phage Antibody Library

The library DNA-transformed TG1 was grown to log phase, infected with M13KO7 helper phage or Hyperphage (Progen, Heidelberg, Germany) and incubated at 37 °C for 30 min. The infected cells were spun down, resuspended in 2xYT medium containing 100 μg/mL ampicillin and 25 μg/mL kanamycin (2YT-AK), and grown overnight with shaking at 30 °C. Phage particles were precipitated, concentrated by polyethylene glycol (PEG)/NaCl (20% PEG 8000, 2.5 M NaCl), and resuspended in phosphate buffered saline (PBS).

### 4.5. Selection of Phage Antibody Library

#### 4.5.1. Solid-Phase Panning

A ninety-six well MaxiSorp^®^ ELISA plate (Nunc, Rochester, NY, USA) was coated with recombinant human VEGF (1 μg/well) in 50 mM sodium hydrogen carbonate (pH 9.6), and blocked with PBS containing 5% skim milk. After washed with PBS, the phage library (10^10^~10^11^ plaque forming units (PFU)) was incubated with the antigen-coated well for 90 min at 37 °C, and washed with 0.05% Tween 20-PBS (PBST). The bound phages were eluted by adding 100 μL triethylamine (100 mM) at 37 °C for 30 min, and the eluate was neutralized with 50 μL Tris buffer (1 M, pH 7.4). The eluate was mixed with 10 mL of exponentially growing TG1 and incubated for 30 min at 37 °C without shaking. The infected bacteria were plated on 2xYT containing 100 μg/mL ampicillin and 2% glucose (2YT-AG) plates and grown at 30 °C overnight. Colonies grown on the plates were scraped and allowed to grow to mid-log phase. The culture was infected with M13KO7 helper phage followed by the procedure described above and used for the next round of selection, with reduced concentrations of VEGF and increased wash stringency after the binding process.

#### 4.5.2. Solution-Phase Panning

The VEGF phage library was pre-incubated with streptavidin-coated paramagnetic beads (Dynabeads M-280) (Invitrogen, Carlsbad, CA, USA) to remove non-specific binders in the library and subjected to 3 rounds of selection in solution with biotinylated human VEGF. The phage library and 50 nM biotin-labeled VEGF were mixed and rotated for 90 min at 37 °C, phages bound to VEGF were captured using 2% skim milk-PBS (MPBS)-blocked Dynabeads. The beads were washed with PBST, 2% MPBS and PBS sequentially. The bound phage was eluted and prepared for the next round of selection followed by the procedure described above.

### 4.6. ELISA Screening of Phage Binders

Individual colonies isolated from each round of selection were inoculated in 96-well plates and rescued with M13KO7 helper phage as described above. For phage ELISA, 96-well MaxiSorp^®^ ELISA plates were coated overnight with 1 μg recombinant human VEGF per well, then blocked with 2% MPBS, and followed by an incubation with phage culture supernatant for 90 min at 37 °C. The plates were washed with PBS, incubated with HRP-conjugated mouse anti-M13 antibody (Amersham Bioscience), developed with 3,3′,5,5′-tetramethylbenzidine (KPL), and stopped by 1 M sulfuric acid. The absorbance at 450 nm was measured with the absorbance at 650 nm used as a background control.

### 4.7. Small-Scale Expression

ScFvs recognizing VEGF were subcloned from phage display vector pCANTAB-5E (Amersham Biosciences) into expression vector pET-27b(+) (Novagen, Madison, WI, USA) via *Nco*I/*Not*I. *E. coli* BL21 were transformed with the expressing plasmids and grown overnight at 37 °C. The scFv expression was induced by 1 mM isopropyl-β-d-thiogalactopyranoside (MDBio, 101-367-93-1) at 30 °C. The bacteria were harvested and lysed in a microfluidizer (Model 110S, Microfluidics, Newton, MA, USA), and the scFvs were purified by HisTrap column (GE, Piscataway, NJ, USA) according the manufacturer’s instructions. For expression of Fab antibodies, TG1 transformation was applied for Fab-pCOMB3X expression. The induction and the purification procedures of Fab antibodies were the same as described above.

For mouse-human chimeric antibody expression, the heavy chain and light chain variable regions were subcloned into expression vector as described previously [46]. Chimeric antibodies were expressed in the FreeStyle 293™ cells (Invitrogen) by transient transfection. Transfection mixtures were generated in separated tubes by adding 37.5 µg of plasmid DNA and 75 µg linear polyethyleneiminie (Polysciences, Warrington, PA, USA) in 150 mM NaCl, respectively. The DNA and polyethylenimine (PEI) solutions were incubated at room temperature for 5 min, and mixed for another 10 min. DNA/PEI mixture was then added to the culture, and incubated for 4 h. After incubation, 15 mL of fresh medium was added and cultured for 5–7 days. The supernatant were harvested for antibody purification by using the Montage Antibody Purification PROSEP-A kit (Millipore, Billerica, MA, USA).

### 4.8. Determination of Antibody Binding Affinity by ELISA

Experiment conditions of antigen coating and washing stringency were as described as phage ELISA. After washing with PBS, wells were incubated with two-fold serial diluted VEGF scFv, Fab or chimeric antibodies in 5% MPBS for 1.5 h at 37 °C. The plates were washed and goat polyclonal anti-human IgG-HRP antibody (1:10,000) (Jackson ImmunoResearch, West Grove, PA, USA) or anti-His antibody (1:5000) (Sigma) was added into each well. The absorbance was measured as described above and the binding affinities of VEGF antibodies were calculated by non-linear regression with Prism software (GraphPad, San Diego, CA, USA).

### 4.9. Epitope Mapping

The alanine mutations were introduced into the wild type sequence of human VEGF by site-directed mutagenesis to generate mutant variants: mutant 1 (F17A, M18A, Y21A, and Y25A), mutant 2 (I46A and K48A), mutant 3 (D63A and L66A), and mutant 4 (M81A, I83A, Q89A, and G92A). All the wild-type and mutant VEGFs were expressed in the FreeStyle 293™ cells by transient transfection. The binding of VEGF antibodies against wild type VEGF or mutants was analyzed by ELISA. The ELISA plates were coated with human VEGF or its mutants (100 µL, 1 µg/mL in coating buffer) overnight at 4 °C. The plates were washed and developed following the procedure of the chimeric antibody ELISA described above.

### 4.10. Determination of Kinetics Rate Constants and Affinity by SPR

Interactions between various anti-VEGF antibodies and VEGF were analyzed by SPR detection using a BIAcore T100 instrument (GE). In a BIAcore flow cell, anti-human IgG (Fc) antibody (GE) was covalently immobilized to a CM5 chip surface to ~10,000 response units (RU) according to the manufacturer’s instructions. Approximately 50–150 RU of the VEGF antibody was captured onto the chip. 0–10 nM of VEGF were measured under a constant flow rate of 40 µL/min using running buffer HBS-EP+ (GE) with a 300 s contact time followed by a 500 s dissociation time. The association (*K*_on_) and dissociation (*K*_off_) phase data were fitted simultaneously to a 1:1 Langumir global model by using the nonlinear data analysis program BIAcore T100 evaluation software (GE).

### 4.11. Proliferation Assay

Various concentrations of anti-VEGF antibodies or Avastin were mixed with 2.5 ng Chinese hamster ovary (CHO) hVEGF in 96-Well Tissue Culture Plates (TPP) and followed by 30 min incubation at room temperature. HUVECs (Cascade Biologics, Portland, OR, USA) were harvested and then resuspended in Medium 200 (Invitrogen) to the density of 8 × 10^4^ cells/mL. An aliquot of 50 μL of this cell culture was added to each well and incubated for 96 h. WST-1 cell proliferation assay reagent (Cell Biolabs, San Diego, CA, USA) was then added and incubated. Colorimetric readings of the absorbance at 450 and 655 nm were measured to derive OD difference (A450 nm–A655 nm) using an ELISA reader. EC_50_ values were calculated using a Four Parameter Logistic (4-PL) model in SigmaPlot 10.0 (Systat, San Jose, CA, USA).

### 4.12. Phosphorylation Assay

HUVECs were grown to 90% confluence with growth factor starvation (2% FBS) at 37 °C for 16 h in a 6-well dish (Greiner bio-one, Kremsmünster, Austria). A mixture containing VEGF (10 ng/mL) and various amounts of test antibodies (0.08, 0.4, 2, 10, and 50 nM) was incubated at 37 °C for 30 min. The VEGF-antibody mixture was added into the culture medium and incubated at room temperature for 10 min. Cell lysates were separated by electrophoresis and analyzed by Western blot with anti-VEGFR2 (1:1000) (Cell Signaling, Beverly, MA, USA) and anti-phospho-VEGFR2 (Tyr1175, 1:1000) (Cell Signaling).

### 4.13. Migration Assay

The migration activity of HUVECs was evaluated using a Transwell assay system (BD Biosciences, San Jose, CA, USA). 5 × 10^4^ cells in 200 μL Medium 200 containing 2% FBS were added to the top chamber, whereas 10 ng/mL migration stimuli VEGF and various concentrations of the test antibodies were added to the lower chamber in 650 μL of the same medium. Cells were allowed to migrate at 37 °C for 22 h. After incubation, the upper surface of the membrane was scraped using a cotton swab to remove the non-migrated cells, and cells on the lower face of the membrane were stained with 0.1% crystal violet at room temperature for 1 h. Cells were counted under microscope at 100× magnification. The cell counts were obtained from three random fields and presented as mean ± SD.

### 4.14. Stable Expression and Tumor Xenograft Assays

To generate anti-VEGF chimeric antibody-expressing stable cell pools, transfection of FreeStyle 293 cells by polyethyleneiminie method was performed as described above. Transfected cells were selected by geneticin (G418) (Sigma). The anti-VEGF antibodies from the stable cell pools were purified as described above and used in the tumor xenograft assays.

COLO 205 cancer cells (5 × 10^6^ cells, from Bioresource Collection and Research Center, Hsinchu, Taiwan) were subcutaneously injected at lateral area on the back of the anesthetized five-week-old female BALB/cAnN.Cg-Foxnlnu/CrlNarl mice. All animal experiments were carried out under the approval of Institutional Animal Care and Use Committee of United Biomedical, Inc., Asia (UBIA) (AT1311, 11 June 2013). When tumors reached a volume greater than the size of 150 mm^3^, the mice were treated intraperitoneally with Avastin or the test antibodies at the dosage of 0.1 mg/kg twice a week over three weeks. The tumor sizes and body weights of the mice were also measured twice weekly. At the end of the study, the mice were sacrificed and the tumors were excised and weighed. The tumor volume was estimated by the formula: volume = ½ × length × width^2^. The tumor growth ratio was calculated by the formula: growth ratio (%) = [(Tumor volume on tumor measuring day)/(Tumor volume on Day 0)] × 100%, where Day 0 means the day drug treatment started.

## References

[1] Blau H.M., Banfi A. (2001). The well-tempered vessel. Nat. Med..

[2] Carmeliet P. (2000). Mechanisms of angiogenesis and arteriogenesis. Nat. Med..

[3] Folkman J. (2007). Angiogenesis: An organizing principle for drug discovery?. Nat. Rev. Drug Discov..

[4] Ferrara N., Kerbel R.S. (2005). Angiogenesis as a therapeutic target. Nature.

[5] Carmeliet P. (2003). Angiogenesis in health and disease. Nat. Med..

[6] Weis S.M., Cheresh D.A. (2011). Tumor angiogenesis: Molecular pathways and therapeutic targets. Nat. Med..

[7] Prewett M., Huber J., Li Y., Santiago A., O’Connor W., King K., Overholser J., Hooper A., Pytowski B., Witte L. (1999). Antivascular endothelial growth factor receptor (fetal liver kinase 1) monoclonal antibody inhibits tumor angiogenesis and growth of several mouse and human tumors. Cancer Res..

[8] Gerber H.-P., Kowalski J., Sherman D., Eberhard D.A., Ferrara N. (2000). Complete inhibition of rhabdomyosarcoma xenograft growth and neovascularization requires blockade of both tumor and host vascular endothelial growth factor. Cancer Res..

[9] Baker C.H., Solorzano C.C., Fidler I.J. (2002). Blockade of vascular endothelial growth factor receptor and epidermal growth factor receptor signaling for therapy of metastatic human pancreatic cancer. Cancer Res..

[10] Boere I.A., Hamberg P., Sleijfer S. (2010). It takes two to tango: Combinations of conventional cytotoxics with compounds targeting the vascular endothelial growth factor-vascular endothelial growth factor receptor pathway in patients with solid malignancies. Cancer Sci..

[11] Ferrara N., Hillan K.J., Novotny W. (2005). Bevacizumab (Avastin), a humanized anti-VEGF monoclonal antibody for cancer therapy. Biochem. Biophys. Res. Commun..

[12] Hurwitz H., Fehrenbacher L., Novotny W., Cartwright T., Hainsworth J., Heim W., Berlin J., Baron A., Griffing S., Holmgren E. (2004). Bevacizumab plus irinotecan, fluorouracil, and leucovorin for metastatic colorectal cancer. N. Engl. J. Med..

[13] Miller K., Wang M., Gralow J., Dickler M., Cobleigh M., Perez E.A., Shenkier T., Cella D., Davidson N.E. (2007). Paclitaxel plus bevacizumab *versus* paclitaxel alone for metastatic breast cancer. N. Engl. J. Med..

[14] Muller Y.A., Chen Y., Christinger H.W., Li B., Cunningham B.C., Lowman H.B., de Vos A.M. (1998). VEGF and the Fab fragment of a humanized neutralizing antibody: Crystal structure of the complex at 2.4 Å resolution and mutational analysis of the interface. Structure.

[15] Marks J.D., Hoogenboom H.R., Bonnert T.P., McCafferty J., Griffiths A.D., Winter G. (1991). By-passing immunization: Human antibodies from V-gene libraries displayed on phage. J. Mol. Biol..

[16] Carter P.J. (2006). Potent antibody therapeutics by design. Nat. Rev. Immunol..

[17] Hoogenboom H.R. (2005). Selecting and screening recombinant antibody libraries. Nat. Biotechnol..

[18] Wiesmann C., Fuh G., Christinger H.W., Eigenbrot C., Wells J.A., de Vos A.M. (1997). Crystal structure at 1.7 Å resolution of VEGF in complex with domain 2 of the Flt-1 receptor. Cell.

[19] Fuh G., Wu P., Liang W.-C., Ultsch M., Lee C.V., Moffat B., Wiesmann C. (2006). Structure-function studies of two synthetic anti-vascular endothelial growth factor Fabs and comparison with the Avastin Fab. J. Biol. Chem..

[20] Holmes K., Roberts O.L., Thomas A.M., Cross M.J. (2007). Vascular endothelial growth factor receptor-2: Structure, function, intracellular signalling and therapeutic inhibition. Cell Signal..

[21] Neufeld G., Cohen T., Gengrinovitch S., Poltorak Z. (1999). Vascular endothelial growth factor (VEGF) and its receptors. FASEB J..

[22] Molek P., Strukelj B., Bratkovic T. (2011). Peptide phage display as a tool for drug discovery: Targeting membrane receptors. Molecules.

[23] Weisser N.E., Hall J.C. (2009). Applications of single-chain variable fragment antibodies in therapeutics and diagnostics. Biotechnol. Adv..

[24] Nelson A.L., Dhimolea E., Reichert J.M. (2010). Development trends for human monoclonal antibody therapeutics. Nat. Rev. Drug Discov..

[25] Bradbury A.R.M., Sidhu S., Dübel S., McCafferty J. (2011). Beyond natural antibodies: The power of *in vitro* display technologies. Nat. Biotechnol..

[26] Zhu Z., Rockwell P., Lu D., Kotanides H., Pytowski B., Hicklin D.J., Bohlen P., Witte L. (1998). Inhibition of vascular endothelial growth factor-induced receptor activation with anti-kinase insert domain-containing receptor single-chain antibodies from a phage display library. Cancer Res..

[27] Kobayashi N., Karibe T., Goto J. (2005). Dissociation-independent selection of high-affinity anti-hapten phage antibodies using cleavable biotin-conjugated haptens. Anal. Biochem..

[28] Santala V., Saviranta P. (2004). Affinity-independent elution of antibody-displaying phages using cleavable DNA linker containing streptavidin beads. J. Immunol. Methods.

[29] Bradbury A.R.M., Marks J.D. (2004). Antibodies from phage antibody libraries. J. Immunol. Methods.

[30] Pini A., Viti F., Santucci A., Carnemolla B., Zardi L., Neri P., Neri D. (1998). Design and use of a phage display library. Human antibodies with subnanomolar affinity against a marker of angiogenesis eluted from a two-dimensional gel. J. Biol. Chem..

[31] Hawkinsov R.E., Winter G. (1992). Cell selection strategies for making antibodies from variable gene libraries: Trapping the memory pool. Eur. J. Immunol..

[32] Rondot S., Koch J., Breitling F., Dübel S. (2001). A helper phage to improve single-chain antibody presentation in phage display. Nat. Biotechnol..

[33] Zhou Y., Goenaga A.-L., Harms B.D., Zou H., Lou J., Conrad F., Adams G.P., Schoeberl B., Nielsen U.B., Marks J.D. (2012). Impact of intrinsic affinity on functional binding and biological activity of EGFR antibodies. Mol. Cancer Ther..

[34] Sullivan L.A., Brekken R.A. (2010). The VEGF family in cancer and antibody-based strategies for their inhibition. MAbs.

[35] Brekken R.A., Overholser J.P., Stastny V.A., Waltenberger J., Minna J.D., Thorpe P.E. (2000). Selective inhibition of vascular endothelial growth factor (VEGF) receptor 2 (KDR/Flk-1) activity by a monoclonal anti-VEGF antibody blocks tumor growth in mice. Cancer Res..

[36] Roland C.L., Dineen S.P., Lynn K.D., Sullivan L.A., Dellinger M.T., Sadegh L., Sullivan J.P., Shames D.S., Brekken R.A. (2009). Inhibition of vascular endothelial growth factor reduces angiogenesis and modulates immune cell infiltration of orthotopic breast cancer xenografts. Mol. Cancer Ther..

[37] Lu D., Jimenez X., Zhang H., Bohlen P., Witte L., Zhu Z. (2002). Selection of high affinity human neutralizing antibodies to VEGFR2 from a large antibody phage display library for antiangiogenesis therapy. Int. J. Cancer.

[38] Sukhramani P.S., Suthar M.P. (2010). VEGF inhibitors for cancer therapy. Int. J. Pharm. Sci. Drug Res..

[39] Hojjat-Farsangi M. (2014). Small-molecule inhibitors of the receptor tyrosine kinases: Promising tools for targeted cancer therapies. Int. J. Mol. Sci..

[40] Niederfellner G., Lammens A., Mundigl O., Georges G.J., Schaefer W., Schwaiger M., Franke A., Wiechmann K., Jenewein S., Slootstra J.W. (2011). Epitope characterization and crystal structure of GA101 provide insights into the molecular basis for type I/II distinction of CD20 antibodies. Blood.

[41] Dalle S., Reslan L., Besseyre de Horts T., Herveau S., Herting F., Plesa A., Friess T., Umana P., Klein C., Dumontet C. (2011). Preclinical studies on the mechanism of action and the anti-lymphoma activity of the novel anti-CD20 antibody GA. Mol. Cancer Ther..

[42] Klein C., Lammens A., Schäfer W., Georges G., Schwaiger M., Mössner E., Hopfner K.-P., Umaña P., Niederfellner G. (2013). Epitope interactions of monoclonal antibodies targeting CD20 and their relationship to functional properties. MAbs.

[43] Desai A.V., El-Bakkar H., Abdul-Hay M. (2014). Novel Agents in the treatment of chronic lymphocytic leukemia: A review about the future. Clin. Lymphoma. Myeloma Leuk..

[44] McCafferty J., Johnson K.S., Kay B.K., Winter J., McCafferty J. (1996). Construction and screening of antibody display libraries. Phage Display of Peptides and Proteins: A Laboratory Manual.

[45] Barbas C.F., Burton D.R., Scott J.K., Silverman G.J. (2001). Phage Display: A Laboratory Manual.

[46] Reff M.E., Carner K., Chambers K.S., Chinn P.C., Leonard J.E., Raab R., Newman R.A., Hanna N., Anderson D.R. (1994). Depletion of B cells *in vivo* by a chimeric mouse human monoclonal antibody to CD20. Blood.

